# Supplementary material for: Epithelioid neoplasm of the spinal cord in a child with spinal muscular atrophy treated with onasemnogene abeparvovec
Source: Mol Ther. 2023 Aug 19;31(10):2991–8. doi: 10.1016/j.ymthe.2023.08.013 (PMC10556221; doi:10.1016/j.ymthe.2023.08.013)
Supplement: Document S1. Figure S1 [file mmc1.pdf]

YMTHE, Volume *31*

Supplemental Information

Epithelioid neoplasm of the spinal cord in a child with spinal muscular atrophy treated with onasemnogene abeparvovec

Laura Retson, Nishant Tiwari, Jennifer Vaughn, Saunder Bernes, P. David Adelson, Keith Mansfield, Silvana Libertini, Brent Kuzmiski, Iulian Alecu, Richard Gabriel, and Ross Mangum

SUPPLEMENTAL MATERIAL

SUPPLEMENTAL FIGURES

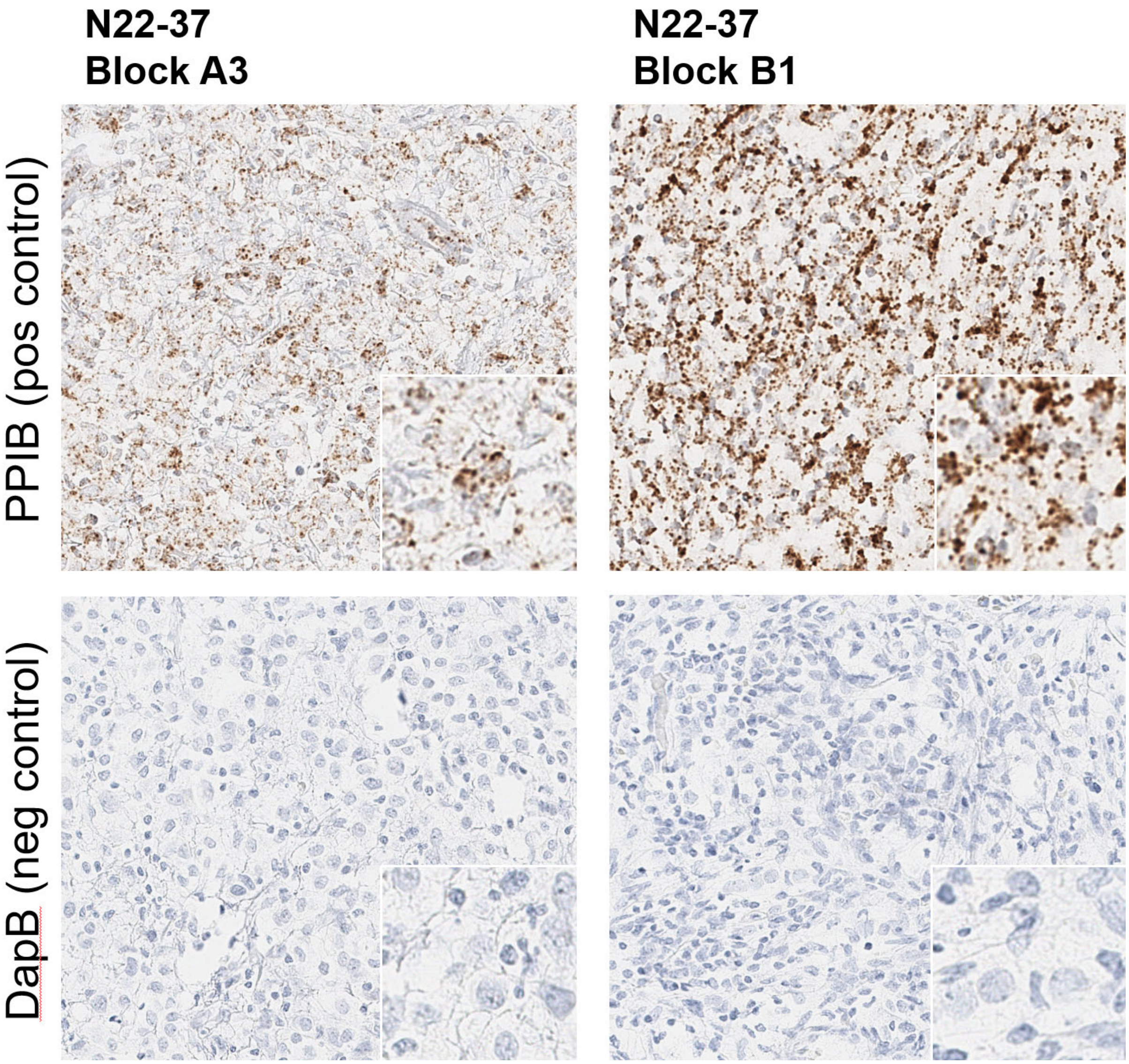

Figure S1. ***In situ*** **hybridization for tissue positive (PPIB) and negative (DapB) controls.**
