# Supplementary material for: Epithelioid neoplasm of the spinal cord in a child with spinal muscular atrophy treated with onasemnogene abeparvovec
Source: Mol Ther. 2023 Aug 19;31(10):2991–8. doi: 10.1016/j.ymthe.2023.08.013 (PMC10556221; doi:10.1016/j.ymthe.2023.08.013)
Supplement: Document S2. Article plus supplemental information [file mmc2.pdf]

Molecular Therapy
Original Article

Epithelioid neoplasm of the spinal cord in a child with spinal muscular atrophy treated with onasemnogene abeparvovec

Laura Retson,[1] Nishant Tiwari,[2] Jennifer Vaughn,[3] Saunder Bernes,[4] P. David Adelson,[5] Keith Mansfield,[6] Silvana Libertini,[6] Brent Kuzmiski,[6] Iulian Alecu,[7] Richard Gabriel,[8] and Ross Mangum[1,9,10,11]

[1]Center for Cancer and Blood Disorders, Phoenix Children's Hospital, Phoenix, AZ 85016, USA; [2]Pathology & Laboratory Medicine, Phoenix Children's Hospital, Phoenix, AZ 85016, USA; [3]Department of Radiology, Phoenix Children's Hospital, Phoenix, AZ 85016, USA; [4]Department of Neurology, Phoenix Children's Hospital, Phoenix, AZ 85016, USA; [5]Department of Neurosurgery, Rockefeller Neuroscience Institute, West Virginia University, Morgantown, WV 26506, USA; [6]Novartis Institutes for BioMedical Research, Cambridge, MA 02139, USA; [7]Novartis Pharmaceuticals, 4056 Basel, Switzerland; [8]ProtaGene CGT GmbH, 74076 Heidelberg, Germany; [9]Department of Child Health, University of Arizona College of Medicine, Phoenix, AZ, USA; [10]Creighton University School of Medicine, Phoenix, AZ 85012, USA; [11]Mayo Clinic Alix School of Medicine, Phoenix, AZ 85054, USA

Spinal muscular atrophy is an autosomal recessive disease resulting in motor neuron degeneration and progressive life-limiting motor deficits when untreated. Onasemnogene abeparvovec is an adeno-associated virus serotype 9-based gene therapy that improves survival, motor function, and motor milestone achievement in symptomatic and presymptomatic patients. Although the adeno-associated virus genome is maintained as an episome, theoretical risk of tumorigenicity persists should genomic insertion occur. We present the case of a 16-month-old male with spinal muscular atrophy who was diagnosed with an epithelioid neoplasm of the spinal cord approximately 14 months after receiving onasemnogene abeparvovec. *In situ* hybridization analysis detected an onasemnogene abeparvovec nucleic acid signal broadly distributed in many but not all tumor cells. Integration site analysis on patient formalin-fixed, paraffin-embedded tumor samples failed to detect high-confidence integration sites of onasemnogene abeparvovec. The finding was considered inconclusive because of limited remaining tissue/DNA input. The improved life expectancy resulting from innovative spinal muscular atrophy therapies, including onasemnogene abeparvovec, has created an opportunity to analyze the long-term adverse events and durability of these therapies as well as identify potential disease associations that were previously unrecognized because of the premature death of these patients.

INTRODUCTION

Spinal muscular atrophy (SMA) is an autosomal recessive disease characterized by deletion or mutation in the survival motor neuron 1 gene (*SMN1*). The resulting decrease in survival motor neuron (SMN) protein expression leads to progressive death of lower motor neurons in the spinal cord. Two almost identical copies of the *SMN* gene, *SMN1* and *SMN2*, are present in humans in variable copy numbers on chromosome 5q13. Although *SMN1* expresses full-length SMN protein, the *SMN2* transcripts lack exon 7, which is predominantly but not always skipped because of a point mutation in the splicing enhancer in exon 7 (C-to-T transition at codon 280).[1] Consequently, most *SMN2*-derived transcripts (up to 85%) are truncated and unstable. SMA severity is largely dependent on the number of copies of *SMN2*.[2] Patients with greater numbers of *SMN2* copies generally have milder disease that manifests later and progresses more slowly compared with patients with fewer *SMN2* copies ($\leq$2 copies).[3] Without treatment, infants with severe, infantile-onset SMA develop hypotonia and muscle weakness so that they fail to achieve key motor milestones (e.g., sitting independently), require mechanical ventilation, and eventually succumb to early death secondary to respiratory failure.[4–6]

Therapeutic options for SMA have evolved from a purely supportive care approach to include three US Food and Drug Administration (FDA)-approved disease-modifying treatments that improve life expectancy with motor outcomes not observed in natural history.[7] Two of these therapies increase SMN protein production derived from *SMN2*, and both require lifelong intrathecal (nusinersen) or oral (risdiplam) dosing.[8,9] Onasemnogene abeparvovec is a gene therapy based on a recombinant adeno-associated virus serotype 9 (AAV9) expressing the human SMN protein. Because of the persistence of AAV as an episome,[10] a single intravenous administration is sufficient for a long-lasting therapeutic effect.[11] For symptomatic and presymptomatic patients treated with intravenous onasemnogene abeparvovec, survival, motor function, and motor milestone achievement were improved compared with natural history.[12–17]

Increased survival has created opportunities to analyze the long-term adverse events (AEs) and durability of these therapies for SMA as well

Received 7 June 2023; accepted 17 August 2023;
https://doi.org/10.1016/j.ymthe.2023.08.013.

Correspondence: Ross Mangum, Center for Cancer and Blood Disorders, Phoenix Children's Hospital, 1919 E. Thomas Road, Phoenix, AZ 85016, USA.
E-mail: mmangum@phoenixchildrens.com

This is an open access article under the CC BY license (http://creativecommons.org/licenses/by/4.0/).

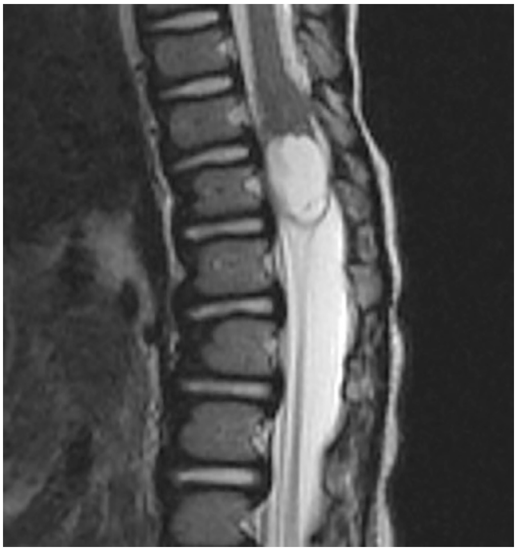

Figure 1. Magnetic resonance imaging analysis

Shown is sagittal T2-weighted magnetic resonance imaging of the thoracolumbar spine, demonstrating an expansile, mixed solid and cystic mass centered in the conus at T12.

as identify potential disease associations that were previously unrecognized because of the premature death of these patients. Here, we present the case of a 16-month-old male infant with SMA who was diagnosed with an epithelioid neoplasm of the spinal cord approximately 14 months after receiving a one-time infusion of onasemnogene abeparvovec.

RESULTS

A white male at risk for SMA based on family history (one older sister who became symptomatic at 14 months of age and treated with nusinersen and another older sister diagnosed at 10 days of age and treated with nusinersen) and the presence of only three copies of *SMN2* was treated with intravenous onasemnogene abeparvovec at 2 months of age prior to symptom onset. The patient's motor development and neurological evaluation were normal 1 month prior to presentation, with no evidence of weakness, abnormal tone, or abnormalities of posture or reflexes. At 16 months of age, the patient presented with 1 week of progressive reduced motor movements, inability to stand, increased fussiness, and urinary retention. Magnetic resonance imaging of the neuroaxis demonstrated a $1.4 \times 1.3 \times 2.2$ cm^3 mixed solid enhancing and cystic/necrotic, expansile, intramedullary lesion centered in the conus medullaris at T12 (Figure 1). No radiographic evidence of tumor dissemination to the brain or spine was present. The radiologic appearance was not characteristic of the more common tumoral entities encountered at this location, such as glial tumors.

The patient underwent T10 to L1 osteoplastic laminoplasty for microscopic excision of the spinal cord tumor. The procedure was challenging because of the age of the child, intertwining of the tumor, and anatomy of the pathologic process. Somatosensory evoked potentials did not change, but intraoperative motor evoked potentials and rectal electromyography

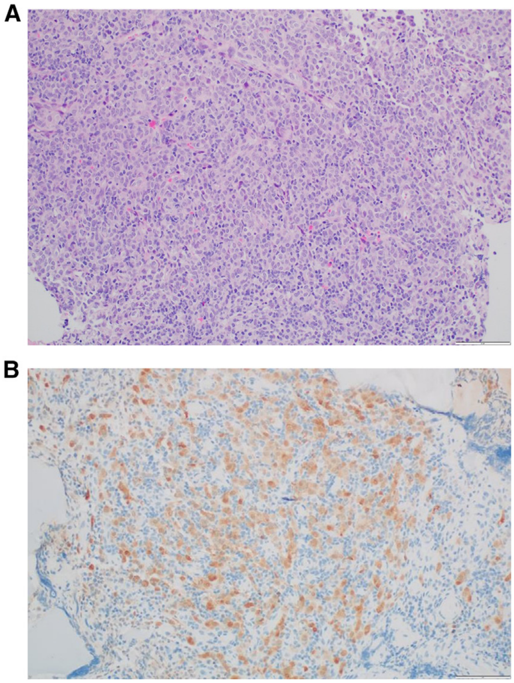

Figure 2. Surgical pathology of resected spinal tumor

(A) Histology demonstrating atypical cells with epithelioid morphology and rare mitosis as well as numerous small infiltrating lymphocytes.

(B) Nuclear and cytoplasmic positivity for S100 in large atypical cells by immunohistochemistry.

improved with decompression. The patient exhibited prompt postoperative improvement of rectal tone, urinary retention, and lower extremity motor weakness with increased ability to bear weight and eventually full recovery of the lower-extremity motor weakness.

Follow-up imaging has been stable without evidence of residual or recurrent tumor 6 months post resection. At 30 months of age, the patient continues to meet all motor and developmental milestones. He eats and drinks independently without aspiration and requires no respiratory assistive devices or supplemental oxygen.

Surgical pathology demonstrated a low-grade epithelioid neoplasm with nonspecific myoepithelial differentiation (Figure 2A). Immunohistochemistry demonstrated S100 and partial cytokeratin AE1/AE3 positivity (Figure 2B). No other immunophenotypic markers of significance were identified. Most notably, the tumor was found to be negative for Brachyury, CD68, SRY-box transcription factor 10 (SOX10), HMB45, glial fibrillary acidic protein, oligodendrocyte transcription factor 2, anaplastic lymphoma kinase, desmin, CD117, synaptophysin, and neurofilament. The tumor cells demonstrated an increase in admixed lymphocytes and plasma cells and increased programmed death ligand 1 (PD-L1) expression.

Comprehensive whole-exome and transcriptome testing identified no definite mutations of pathological significance. Analysis of RNA

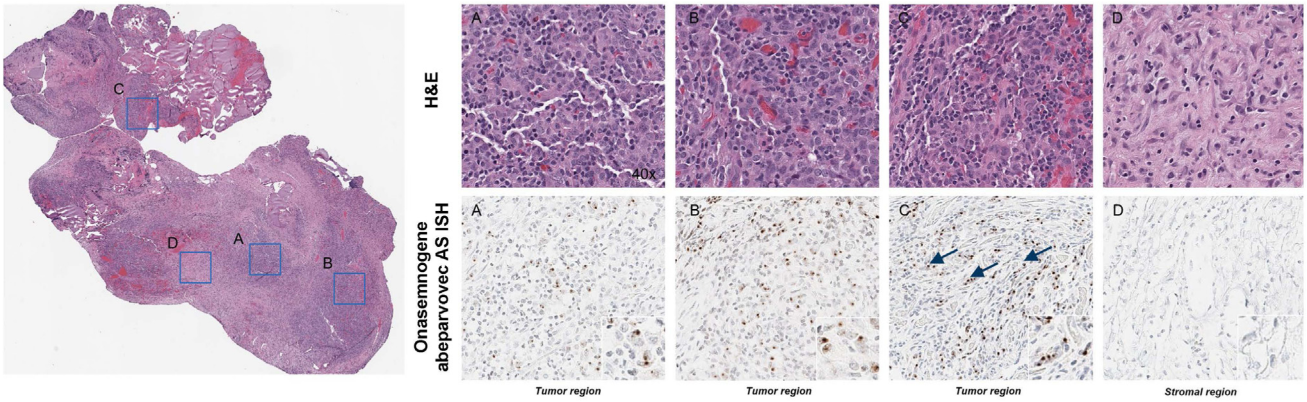

Figure 3. *In situ* hybridization (ISH) for onasemnogene abeparvovec vector nucleic acid in FFPE tissue sections using an AS RNAscope probe
The signal is apparent as discrete intranuclear DAB (brown) foci (arrows) and highlighted in the insets. Panels on the right are 40× magnifications of blue boxed regions. H&E, hematoxylin and eosin.

expression demonstrated relative overexpression of *PTCH1* and *GLI1*, suggesting Sonic Hedgehog pathway activation (data not shown). Methylation profiling demonstrated no match to any of the known entities in the central nervous system or other soft tissue lesions.

In situ hybridization (ISH) analysis using probes specific for onasemnogene abeparvovec detected a vector nucleic acid signal broadly distributed in many, but not all, tumor cells present in tissue sections obtained from two individual tissue samples (Figure 3). The signal was absent from adjacent non-neoplastic stroma and infiltrating immune cells. ISH analysis of the positive and negative controls is presented in Figure S1.

To evaluate whether and where the viral vector integrated into host genomic DNA, shearing extension primer tag selection ligation-mediated polymerase chain reaction (S-EPTS/LM-PCR) was performed on DNA extracted from formalin-fixed, paraffin-embedded (FFPE) tumor samples. Integration site analysis of the patient samples failed to detect high-confidence integration sites of onasemnogene abeparvovec; however, the analysis was considered inconclusive because of limited remaining tissue and DNA input. A total of five unique exactly mappable integration sites were detected by S-EPTS/LM-PCR in three analyzed samples (Table 1; Figure 4). The majority of the raw sequencing reads contained sequences mapping to the vector but were lacking genomic sequences reflecting an integration event (data not shown). It is also worth noting that the primers used for the S-EPTS/LM-PCR were upstream of the inverted terminal repeat (ITR) region (in the bovine growth hormone polyadenylation [bGHpA] region). The intact ITR has a length of 145 bp, and the reported average read length (58 bp) was insufficient to reach the genomic DNA. Also, the obtained integration sites were considered to be low confidence because each was only represented by one sequencing read, and none of the integration sites were reproducible across the different samples (Table 1). Thus, integration site analysis of the patient samples failed to detect high-confidence integration sites for onasemnogene abeparvovec.

DISCUSSION

This represents the first published case of a patient with SMA treated with gene therapy who later developed an epithelioid neoplasm of the spinal cord. The phenotypic manifestations of SMA vary substantially, from extreme muscle weakness at birth or during infancy resulting in early respiratory failure and death within 2 years (SMA types 0 and 1) to late-onset symptoms of mild muscle weakness with normal life expectancy (SMA type 4).[18] The variability in SMA disease severity depends largely on the *SMN2* copy number.[2,3] Based on confirmatory cord blood genetic testing demonstrating homozygous deletion of *SMN* on chromosome 5q and three copies of the *SMN2* gene, it was determined that our patient was at risk for development of symptomatic SMA. In the past, most SMA patients were treated after symptom onset; however, our patient was treated with onasemnogene abeparvovec at approximately 8 weeks of age, prior to symptom onset.

Onasemnogene abeparvovec, which was approved by the FDA in 2019, is an AAV9-based gene therapy that delivers the human *SMN* gene to restore expression of full-length SMN protein.[18] The patient highlighted here is currently 30 months post onasemnogene abeparvovec infusion and remains fully ambulatory without signs of motor weakness following removal of the spinal tumor. The patient has

Table 1. Overview of the sequencing results and integration sites retrieved

Sample	Sample type	DNA input (ng)	Sorted reads	Unique IS	Sequence count/IS
C1	FFPE scrolls	4.9	212,145	2	1
B1	FFPE slides	55.8	203,295	2	1
A3	FFPE slides	45.0	256,594	1	1
pSMN	control	1.0	221,970	5	1
hgDNA	PBMC	57.0	3	0	N/A

FFPE, formalin-fixed, paraffin-embedded; IS, integration site; pSMN, porcine SMN; hgDNA, human genomic DNA; PBMC, peripheral blood mononuclear cell.

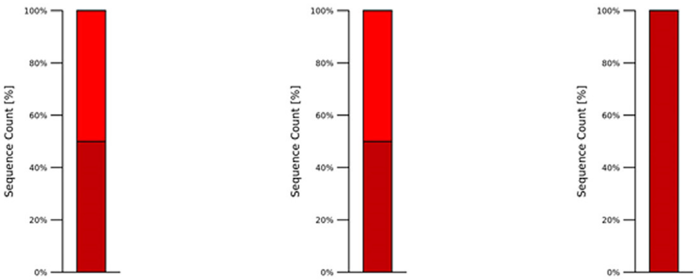

Sample		N22-37-C1			N22-37 B1			N22-37 A3		
Sample Type		FFPE scrolls			FFPE slides			FFPE slides		
Rank		Gene	Freq. [%]	Location	Gene	Freq. [%]	Location	Gene	Freq. [%]	Location
Top1		*RNF219*	50.000	13+78698531	*ERC1*	50.000	12+1286294	*ADCY2*	100	5-7603827
Top2		*TOX2*	50.000	20+44047865	*KIF13A*	50.000	6+17869004	N/A	N/A	N/A
Top3		N/A	N/A	N/A	N/A	N/A	N/A	N/A	N/A	N/A
Top4		N/A	N/A	N/A	N/A	N/A	N/A	N/A	N/A	N/A
Top5		N/A	N/A	N/A	N/A	N/A	N/A	N/A	N/A	N/A
Top6		N/A	N/A	N/A	N/A	N/A	N/A	N/A	N/A	N/A
Top7		N/A	N/A	N/A	N/A	N/A	N/A	N/A	N/A	N/A
Top8		N/A	N/A	N/A	N/A	N/A	N/A	N/A	N/A	N/A
Top9		N/A	N/A	N/A	N/A	N/A	N/A	N/A	N/A	N/A
Top10		N/A	N/A	N/A	N/A	N/A	N/A	N/A	N/A	N/A
#All Other mapp. IS		0	0.000		0	0.000		0	0	
Seq Count 10 strongest		2			2			1		
Seq Count all other mapp. IS		0			0			0		
Total Seq Counts Used		2			2			1		

Figure 4. Cumulative retrieval frequencies of the 10 most prominent integration sites detected in all samples

Sequence count of the 10 most prominent integration sites (ISs; Seq Count 10 Strongest), sequence count of all remaining ISs (Seq Count all other mapp. ISs), and total IS-specific sequence count (Total Seq Count Used) are presented at the bottom for each sample. RefSeq names of genes located closest to the respective IS are given in the table (gene name). Relative sequence count contributions of the 10 most prominent ISs and all remaining mappable ISs are presented (frequency [percent]). The location column comprises chromosome number, sequence orientation (plus or minus), and IS locus (based on human reference genome hg38). FFPE, formalin-fixed, paraffin-embedded; N/A, not available.

never experienced clinical symptoms of SMA, and the motor symptoms that prompted the discovery of his spinal cord tumor lasted only 1 week before excision.

Several studies have highlighted the short-term efficacy and safety of onasemnogene abeparvovec. Mendell et al.[12] reported preliminary data from 15 patients with SMA type 1 treated with a single dose of onasemnogene abeparvovec in an open-label, phase 1 study (START trial; ClinicalTrials.gov: NCT02122952). Patients who received onasemnogene abeparvovec demonstrated improvements in motor function compared with historical controls.[12] Several follow-up studies focusing on the short-term outcomes of the 12 START patients who received the therapeutic dose of onasemnogene abeparvovec confirm effectiveness in key metrics such as pulmonary and nutritional support requirements, achievement of motor milestones, hospitalization rate, and probability of survival compared with natural history.[13,19,20] Subsequent phase 3 trials have further confirmed the efficacy of onasemnogene abeparvovec in patients with SMA type 1 (STR1VE [ClinicalTrials.gov: NCT03306277] and STR1VE-EU [ClinicalTrials.gov: NCT03461289]) and in presymptomatic patients with two or three *SMN2* gene copies at risk for developing SMA (SPR1NT; ClinicalTrials.gov: NCT03505099).[14–17]

Because of the historically poor survival of patients with SMA, very little is known regarding the potential associations between SMA and the risk for developing solid tumors or other malignancies. With improved motor function and longer survival associated with current SMA therapies, there likely will be more opportunities to study long-term health outcomes and concurrent illnesses in this patient population. Although this is the first reported case of an epithelioid neoplasm of the spinal cord associated with SMA, there is one documented case of a 33-year-old female with SMA type 4 who presented with progressive neck pain radiating to the arms and was diagnosed with cervical-thoracic spinal cord ependymoma.[21] In addition, alveolar rhabdomyosarcoma (ARMS) of the forearm has been reported in two patients with SMA (types 2 and 3a).[22] Despite demonstrating early signs of motor dysfunction that prompted their SMA diagnoses as toddlers, both patients survived into their teenage years but died relatively shortly after their ARMS diagnoses because of related complications. Other reported cases of concurrent malignancies diagnosed in patients with SMA include an 11-year-old male with SMA type 3 and metastatic ARMS of the ankle[23] and a 4-month-old male presenting with a periumbilical mass and hypotonia simultaneously diagnosed with neuroblastoma and SMA type 1.[24]

Table 2. Number of predicted copies of integrated vectors in the analyzed samples

					Random integration[d]			
Block	DNA, ng	Diploid genomes corresponding to input[a]	Tumor cells (%)[b]	VCN[c]	1.00%	0.10%	0.01%	Clonal integration[e]
A3	45	6,300	50%	1	31.5	3.15	0.315	3,150
B1	57	7,980	25%	1	19.95	1.995	0.1995	1,995
C1	5	700	75%	1	5.25	0.525	0.0525	525

AAV, adeno-associated virus; H&E, hematoxylin and eosin; LOD, limit of detection; VCN, vector copy number. Example for block A3: 45 ng of DNA corresponded to 6,300 cells (because each human cell contains ∼7.2 pg of DNA). Assuming that 50% of this sample contains tumor cells (as suggested by ISH) and has a VCN of 1, and assuming that integration happens randomly in 1% of the tumor cells, 31 different copies of integrated vector could be expected in the sample. This is likely below the LOD, especially if the integration rate was 0.1% or 0.01%. However, if all cells were derived from the same integrated clone, then 3,150 integrated vector copies (i.e., with the same integration site) would be expected. This is likely above the LOD.

[a]Average weight of DNA contained in one human diploid genome is 7.2 pg.

[b]Percentage tumor cells in sample estimated on H&E-stained serial sections.

[c]Estimated VCN vector copy number in tumor cells based on *in situ* hybridization.

[d]Rate of random integration per VCN from published literature.[8,37–39]

[e]Predicted AAV vector sequence number if clonal integration present in tumor cells.

One advantage of AAV-based gene therapies is that the viral vector primarily persists as an episome following transduction because of a lack of Rep-mediated active integration.[11] Because the efficiency of recombinant AAV (rAAV) integration into the host cell genome is low, the risk of insertional mutagenesis is reduced compared with other viral vectors. However, spontaneous integration into double-stranded breaks is possible.[25] In mice, rAAV-induced genotoxicity has been reported to contribute to hepatocellular carcinoma (HCC) formation,[25–29] and the frequency of insertional mutagenesis may be related to age at dosing, dose, and the enhancer-promoter used.[25] No such events have been reported in nonhuman primates, which may be related to the relatively short-term observations reported to date. However, AAV-related tumorigenesis has also not been reported in long-term studies (up to 10 years) in dog models, where only clonality was observed.[25] At present, AAV-induced tumorigenesis also remains a theoretical risk in humans.[30] One case of HCC in an adult treated with etranacogene dezaparvovec, an AAV5-based gene therapy for hemophilia B, has been reported.[31] Subsequent analysis revealed a low rate of AAV integration (0.027%) without evidence of clonal expansion as well as the presence of several risk factors that may have predisposed the patient to HCC.

In the present study, ISH analyses indicate the presence of the vector nucleic acid within many but not all tumor cells, though the probe cannot distinguish episomal from integrated vector DNA.

Integration site analyses of available patient samples failed to detect high confidence integration sites for onasemnogene abeparvovec. However, it is not possible to exclude that integration sites may have been missed because of the low DNA input, which was far below the optimal value for analysis (5–57 ng/sample instead of 1.5–3 μg). The average short read length may be a direct consequence of the limitations because of the low input material. Also, partial vector integration that lacks the primer binding sites (bGHpA) would not be detected by the applied method. For future studies, alternative methods, such as target enrichment sequencing (TES), could be considered. As opposed to S-EPTS/LM-PCR, which relies on the presence of intact primer binding sites, TES has the advantage of possibly detecting integration sites originating from partially deleted vectors without affecting the limit of detection, as recently demonstrated by Oziolor et al.[32]

This case illustrates the challenges incurred when limited FFPE material is available. The use of histological slides to further define the tumor type and investigate potential precision targets for patient management were prioritized. In instances when adjacent normal tissue is available, the analysis of histological slides may further assist tumor identification.

Table 2 presents the number of predicted copies of integrated vector that would have been present if random integration had occurred in a preexisting tumor mass or if clonal integration had occurred prior to cellular transformation. If integration had occurred in a preexisting tumor mass, then the number of random integration events present would have been extremely low and likely below the limitation of the assay to detect them. In contrast, if clonal integration had resulted prior to cellular transformation, then each tumor cell in the sample would have contained the same integration event, resulting in substantially greater integrated vector copy number. Thus, if clonal integration had driven oncogenesis, then the predicted number of integrated vector sequences in the sample would have been much greater, likely allowing detection of a single clonal site present in all tumor cells. With the current available data, there is no indication of clonal expansion anticipated in cases of oncogenesis driven by AAV vector integration. However, given the assay limitations because of the low DNA input used and the inability to detect random integration events, it cannot be definitively excluded that a dominant insertional site escaped detection.

The increased tumor expression of PD-L1 observed in the case presented here has interesting implications for the tumorigenesis of solid tumors in patients with SMA. In human cancers, increased PD-L1

expression has been correlated with DNA damage.[33] Similarly, key cellular signaling and biochemical pathways associated with DNA damage and impaired DNA repair are activated in patients with SMA.[34] Thus, increased PD-L1 expression could either be secondary to DNA breakdown associated with tumor development or could be related to the SMA disease course. Regardless of the etiology driving PD-L1 expression, this finding carries certain clinical implications. For example, this patient may benefit from routine surveillance screening for other PD-L1-associated cancers. Likewise, specific PD-L1 genetic testing may be indicated for certain family members, including the patient's two siblings with SMA, to determine the potential increased risk for development of secondary malignancies. Further research is needed to determine whether this patient's increased PD-L1 expression could be targeted for PD-L1 inhibition with agents such as pembrolizumab or nivolumab if he experiences recurrence of a spinal tumor that is not favorable for surgical resection.

MATERIALS AND METHODS

Tissue samples

The patient tumor sample was collected and processed in FFPE blocks at the Phoenix Children's Hospital (PCH, Phoenix, AZ, USA). FFPE slides and scrolls were generated from the blocks for ISH analysis and integration site analysis, respectively.

DNA and RNA sequencing analysis

Whole-exome and transcriptome sequencing was performed at Caris Life Sciences (Phoenix, AZ, USA).

ISH

ISH analysis of tissue sections from two FFPE blocks was conducted to detect AAV antisense (AS) and sense (S) sequences as well as Hs-PPIB (peptidylprolyl isomerase B [cyclophilin B], positive control and tissue quality control) and DapB (negative control) genes using reagents and equipment supplied by Advanced Cell Diagnostics (ACDBio; Hayward, CA, USA) and Ventana Medical Systems (Roche, Tucson, AZ, USA). The following ISH RNAscope probes were designed by ACDBio and do not cross-hybridize to the human genome: Hs-PPIB (catalog number 313909), DapB (catalog number 312039), phSMN AS (catalog number 1141319-C1), and phSMN S (catalog number 1141309-C1). Positive PPIB and negative DapB control probe sets were included to ensure mRNA quality and specificity, respectively. The hybridization method followed protocols established by ACDBio and Ventana Medical Systems using a 3,3′-diaminobenzidine (DAB) chromogen. Briefly, 4- 5-μm sections were baked at 60°C for 60 min and used for hybridization. The deparaffinization and rehydration protocol was performed using a Tissue-Tek DR5 stainer (Sakura Finetek USA, Torrance, CA, USA) with the following steps: three times xylene for 3 min each, two times 100% alcohol for 3 min, and air drying for 5 min. Offline manual pretreatment in 1× retrieval buffer at 98°C–104°C was performed for 60 min. Optimization was performed by first evaluating the PPIB and DapB hybridization signal and subsequently using the same conditions for all slides. Following pretreatment, the slides were transferred to a Ventana Medical Systems Ultra autostainer to complete the ISH procedure, including protease pretreatment, hybridization at 43°C for 2 h, followed by amplification, and detection with horseradish peroxidase and hematoxylin counterstain. Archived normal and neoplastic human control tissue samples from non-dosed individuals served as additional negative control material. Engineered HEK293 cells containing onasemnogene abeparvovec vector sequence as well as tissue from onasemnogene abeparvovec-dosed cynomolgus macaques served as positive control material. Hematoxylin and eosin-stained sections were used to confirm tumor presence. Slides were scanned at 20–40× using an Aperio (Leica Biosystems, Deer Park, IL, USA) AT2 scanner.

DNA extraction from FFPE samples for S-EPTS/LM-PCR

DNA was extracted by ProtaGene (Heidelberg, Germany) from each FFPE sample separately using the GeneRead DNA FFPE Kit (QIAGEN, Hilden, Germany) according to the manufacturer's instructions. Briefly, proteins in the samples were digested using Proteinase K for 1 h. After heating to remove crosslinks, artificially induced uracils that are typically from DNA obtained from FFPE samples were removed from the DNA by uracil-N-glycosylase. After binding of DNA to the spin column, residual contaminants, such as salts, were washed away by buffer AW1, buffer AW2, and ethanol. Any residual ethanol, which may interfere with subsequent enzymatic reactions, was removed by an additional centrifugation step. Finally, DNA was eluted in H_2O and stored at −20°C. Because of the low amount of DNA retrieved from each isolation, all DNA obtained from each of three blocks was pooled, and the S-EPTS/LM-PCR analysis was performed as a single sample on each pool. Because of the low amount of genomic DNA isolated from the residual FFPE samples, the assay could only be performed in one replicate per sample with 5–57 ng/sample instead of the 1 μg per replicate (three replicates) of genomic DNA generally used.

Integration site analysis

Integration site analysis of tissue sections from three FFPE blocks was conducted by ProtaGene (Heidelberg, Germany) to evaluate onasemnogene abeparvovec integration in the genomic DNA. This method first employs S-EPTS/LM-PCR for amplification and sequencing of unknown genomic sequences flanking integrated vector DNA coupled with next-generation sequencing and subsequent semi-automated data processing and identification of nearby genes.

All available genomic DNA per sample was sheared to a median length of 500 bp using the M220 instrument (Covaris, Woburn, MA, USA). Primer extension was performed using a bovine growth hormone polyadenylation signal-specific biotinylated primer: 5′-GCATCGCATT GTCTGAGTAGG -3'. The extension product was again purified, followed by magnetic capture of the biotinylated DNA for at least 60 min and two washing steps with 100 μL H_2O. The captured DNA was ligated to linker cassettes, including a molecular barcode. The ligation product was amplified in a first exponential PCR using the following biotinylated vector- and linker cassette-specific primers: 5′-GACCC GGGAGATCTGAATTC-3′ and 5′-GTAGGTGTCATTCTATTCTG

GG-3'. Biotinylated PCR products were magnetically captured and washed, and half of this eluate served as a template for amplification in a second exponential PCR step with the following primers to allow deep sequencing by MiSeq technology (Illumina, San Diego, CA, USA) after purification: 5′-GGAAGACAATAGCAGGCATG-3' (vector specific) and 5′-AGTGGCACAGCAGTTAGG-3' (linker cassette specific). Preparation for deep sequencing was done as described previously.[35,36] DNA double barcoding was applied to allow parallel sequencing of multiple samples in a single sequencing run while minimizing sample cross-contamination.

Control samples included vector (porcine SMN) spiked in human genomic DNA (positive control sample for PCR amplification) and human genomic DNA (negative control sample). Raw sequence data were filtered according to sequence quality, and only sequences demonstrating 100% identity in both molecular barcodes (linker cassette and sequencing barcodes) were further analyzed using Genome Integration Site Analysis Pipeline (GENE-IS) v.1.6.[37] In brief, sequences were trimmed (vector- and linker cassette-specific parts removed) and aligned to the human genome hg38, while nearby genes and other integrating features were annotated as described previously.[36]

Conclusion

We presented the case of a 16-month-old male at risk for SMA treated presymptomatically with onasemnogene abeparvovec at 8 weeks of age who was found to have an epithelioid neoplasm of the spinal cord. Integration site analyses of available patient samples failed to detect integration sites for onasemnogene abeparvovec. However, it is not possible to definitively exclude that integration sites may have been missed because of the low DNA input. The improved life expectancy resulting from innovative SMA therapies has created an opportunity to better understand whether patients with SMA may be at increased risk for the development of certain malignancies. It also permits monitoring the long-term safety profile of onasemnogene abeparvovec. Further research is needed to better understand these associations, if any, and to establish appropriate tumor surveillance protocols for patients with SMA.

DATA AND CODE AVAILABILITY

All research materials and data reported in this article are available upon request to the authors.

SUPPLEMENTAL INFORMATION

Supplemental information can be found online at https://doi.org/10.1016/j.ymthe.2023.08.013.

ACKNOWLEDGMENTS

Medical writing and editorial support were provided by Marjet Heitzer, PhD, of Kay Square Scientific (Newtown Square, PA, USA). This support was funded by Novartis Gene Therapies, Inc.

AUTHOR CONTRIBUTIONS

R.M. conceived, designed, and supervised the case study. R.M., L.R., I.A., and K.M. performed data collection and analysis. L.R., R.M., and K.M. wrote the first draft of the paper. All authors reviewed and approved the final draft for submission.

DECLARATION OF INTERESTS

L.R., N.T., J.V., S.B., P.D.A., R.G., and R.M. have no funding or conflicts of interest to disclose. K.M., B.K., S.L., and I.A. are employees of Novartis and own stock or other holdings.

REFERENCES

1. Lorson, C.L., Hahnen, E., Androphy, E.J., and Wirth, B. (1999). A single nucleotide in the SMN gene regulates splicing and is responsible for spinal muscular atrophy. Proc. Natl. Acad. Sci. USA *96*, 6307–6311. https://doi.org/10.1073/pnas.96.11.630.
2. Mailman, M.D., Heinz, J.W., Papp, A.C., Snyder, P.J., Sedra, M.S., Wirth, B., Burghes, A.H.M., and Prior, T.W. (2002). Molecular analysis of spinal muscular atrophy and modification of the phenotype by SMN2. Genet. Med. *4*, 20–26. https://doi.org/10.1097/00125817-200201000-00004.
3. Calucho, M., Bernal, S., Alías, L., March, F., Venceslá, A., Rodríguez-Álvarez, F.J., Aller, E., Fernández, R.M., Borrego, S., Millán, J.M., et al. (2018). Correlation between SMA type and SMN2 copy number revisited: An analysis of 625 unrelated Spanish patients and a compilation of 2834 reported cases. Neuromuscul. Disord. *28*, 208–215. https://doi.org/10.1016/j.nmd.2018.01.003.
4. Finkel, R.S., McDermott, M.P., Kaufmann, P., Darras, B.T., Chung, W.K., Sproule, D.M., Kang, P.B., Foley, A.R., Yang, M.L., Martens, W.B., et al. (2014). Observational study of spinal muscular atrophy type I and implications for clinical trials. Neurology *83*, 810–817. https://doi.org/10.1212/WNL.000000000000074.
5. Kolb, S.J., Coffey, C.S., Yankey, J.W., Krosschell, K., Arnold, W.D., Rutkove, S.B., Swoboda, K.J., Reyna, S.P., Sakonju, A., Darras, B.T., et al. (2017). Natural history of infantile-onset spinal muscular atrophy. Ann. Neurol. *82*, 883–891. https://doi.org/10.1002/ana.25101.
6. Mercuri, E., Lucibello, S., Perulli, M., Coratti, G., de Sanctis, R., Pera, M.C., Pane, M., Montes, J., de Vivo, D.C., Darras, B.T., et al. (2020). Longitudinal natural history of type I spinal muscular atrophy: a critical review. Orphanet J. Rare Dis. *15*, 84. https://doi.org/10.1186/s13023-020-01356-1.
7. Mercuri, E. (2021). Spinal muscular atrophy: from rags to riches. Neuromuscul. Disord. *31*, 998–1003. https://doi.org/10.1016/j.nmd.2021.08.009.
8. (2016). Spinraza [package Insert] (Biogen), updated 2020 June; cited 2022 May 7. https://www.spinraza.com/content/dam/commercial/spinraza/caregiver/enus/pdf/spinraza-prescribing-information.pdf.
9. (2020). Evrysdi [package Insert] (Genentech), updated 2022 May; cited 2022 May 7. https://www.gene.com/download/pdf/evrysdiprescribing.pdf.
10. Ehrhardt, A., Xu, H., and Kay, M.A. (2003). Episomal persistence of recombinant adenoviral vector genomes during the cell cycle *in vivo*. J. Virol. *77*, 7689–7695. https://doi.org/10.1128/jvi.77.13.7689–7695.2003.
11. (2021). Zolgensma [package Insert] (Novartis Gene Therapies, Inc.), [updated 2023 February; cited 2023 April 14. https://www.novartis.us/sites/www.novartis.us/files/zolgensma.pdf.
12. Mendell, J.R., Al-Zaidy, S., Shell, R., Arnold, W.D., Rodino-Klapac, L.R., Prior, T.W., Lowes, L., Alfano, L., Berry, K., Church, K., et al. (2017). Single-dose gene-replacement therapy for spinal muscular atrophy. N. Engl. J. Med. *377*, 1713–1722. https://doi.org/10.1056/NEJMoa1706198.
13. Al-Zaidy, S.A., Kolb, S.J., Lowes, L., Alfano, L.N., Shell, R., Church, K.R., Nagendran, S., Sproule, D.M., Feltner, D.E., Wells, C., et al. (2019). AVXS-101 (onasemnogene abeparvovec) for SMA1: comparative study with a prospective natural history cohort. J. Neuromuscul. Dis. *6*, 307–317. https://doi.org/10.3233/JND-190403.
14. Day, J.W., Finkel, R.S., Chiriboga, C.A., Connolly, A.M., Crawford, T.O., Darras, B.T., Iannaccone, S.T., Kuntz, N.L., Peña, L.D.M., Shieh, P.B., et al. (2021). Onasemnogene abeparvovec gene therapy for symptomatic infantile-onset spinal muscular atrophy in patients with two copies of SMN2 (STR1VE): an open-label, single-arm,

multicentre, phase 3 trial. Lancet Neurol. *20*, 284–293. https://doi.org/10.1016/S1474-4422(21)00001-6.

15. Mercuri, E., Muntoni, F., Baranello, G., Masson, R., Boespflug-Tanguy, O., Bruno, C., Corti, S., Daron, A., Deconinck, N., Servais, L., et al. (2021). Onasemnogene abeparvovec gene therapy for symptomatic infantile-onset spinal muscular atrophy type 1 (STR1VE-EU): an open-label, single-arm, multicentre, phase 3 trial. Lancet Neurol. *20*, 832–841. https://doi.org/10.1016/S1474-4422(21)00251-9.
16. Strauss, K.A., Farrar, M.A., Muntoni, F., Saito, K., Mendell, J.R., Servais, L., McMillan, H.J., Finkel, R.S., Swoboda, K.J., Kwon, J.M., et al. (2022). Onasemnogene abeparvovec for presymptomatic infants with two copies of SMN2 at risk for spinal muscular atrophy type 1: the Phase III SPR1NT trial. Nat. Med. *28*, 1381–1389. https://doi.org/10.1038/s41591-022-01866-4.
17. Strauss, K.A., Farrar, M.A., Muntoni, F., Saito, K., Mendell, J.R., Servais, L., McMillan, H.J., Finkel, R.S., Swoboda, K.J., Kwon, J.M., et al. (2022). Onasemnogene abeparvovec for presymptomatic infants with three copies of SMN2 at risk for spinal muscular atrophy: the Phase III SPR1NT trial. Nat. Med. *28*, 1390–1397. https://doi.org/10.1038/s41591-022-01867-3.
18. Stevens, D., Claborn, M.K., Gildon, B.L., Kessler, T.L., and Walker, C. (2020). Onasemnogene abeparvovec-xioi: gene therapy for spinal muscular atrophy. Ann. Pharmacother. *54*, 1001–1009. https://doi.org/10.1177/1060028020914274.
19. Al-Zaidy, S., Pickard, A.S., Kotha, K., Alfano, L.N., Lowes, L., Paul, G., Church, K., Lehman, K., Sproule, D.M., Dabbous, O., et al. (2019). Health outcomes in spinal muscular atrophy type 1 following AVXS-101 gene replacement therapy. Pediatr. Pulmonol. *54*, 179–185. https://doi.org/10.1002/ppul.24203.
20. Lowes, L.P., Alfano, L.N., Arnold, W.D., Shell, R., Prior, T.W., McColly, M., Lehman, K.J., Church, K., Sproule, D.M., Nagendran, S., et al. (2019). Impact of age and motor function in a phase 1/2a study of infants with SMA type 1 receiving single-dose gene replacement therapy. Pediatr. Neurol. *98*, 39–45. https://doi.org/10.1016/j.pediatrneurol.2019.05.005.
21. Albakr, A., Abbas, F., Al-Jehani, H., and Ammar, A. (2017). Spinal muscular atrophy and ependymoma. Saudi J. Med. Med. Sci. *5*, 163–166. https://doi.org/10.4103/1658-631X.204857.
22. Yaris, N., Kutluk, T., Topaloğlu, H., Akçören, Z., and Büyükpamukçu, M. (2002). Disseminated alveolar rhabdomyosarcoma in a child with spinal muscular atrophy. J. Pediatr. Hematol. Oncol. *24*, 508–509. https://doi.org/10.1097/00043426-200208000-00022.
23. Rudnik-Schöneborn, S., Anhuf, D., Koscielniak, E., and Zerres, K. (2005). Alveolar rhabdomyosarcoma in infantile spinal muscular atrophy: coincidence or predisposition? Neuromuscul. Disord. *15*, 45–47. https://doi.org/10.1016/j.nmd.2004.09.013.
24. Sag, E., Sen, H.S., Haliloglu, G., Yalcin, B., and Kutluk, T. (2015). Neuroblastoma in a patient with spinal muscular atrophy type I: is it just a coincidence? J. Child Neurol. *30*, 1075–1078. https://doi.org/10.1177/0883073814542950.
25. Miller, D.G., Petek, L.M., and Russell, D.W. (2004). Adeno-associated virus vectors integrate at chromosome breakage sites. Nat. Genet. *36*, 767–773. https://doi.org/10.1038/ng1380.
26. Chandler, R.J., LaFave, M.C., Varshney, G.K., Burgess, S.M., and Venditti, C.P. (2016). Genotoxicity in mice following AAV gene delivery: a safety concern for human gene therapy? Mol. Ther. *24*, 198–201. https://doi.org/10.1038/mt.2016.17.
27. Chandler, R.J., Sands, M.S., and Venditti, C.P. (2017). Recombinant adeno-associated viral integration and genotoxicity: insights from animal models. Hum. Gene Ther. *28*, 314–322. https://doi.org/10.1089/hum.2017.009.
28. de Jong, Y.P., and Herzog, R.W. (2021). Liver gene therapy and hepatocellular carcinoma: a complex web. Mol. Ther. *29*, 1353–1354. https://doi.org/10.1016/j.ymthe.2021.03.009.
29. Gil-Farina, I., Fronza, R., Kaeppel, C., Lopez-Franco, E., Ferreira, V., D'Avola, D., Benito, A., Prieto, J., Petry, H., Gonzalez-Aseguinolaza, G., and Schmidt, M. (2016). Recombinant AAV integration is not associated with hepatic genotoxicity in nonhuman primates and patients. Mol. Ther. *24*, 1100–1105. https://doi.org/10.1038/mt.2016.52.
30. Sabatino, D.E., Bushman, F.D., Chandler, R.J., Crystal, R.G., Davidson, B.L., Dolmetsch, R., Eggan, K.C., Gao, G., Gil-Farina, I., Kay, M.A., et al. (2022). Evaluating the state of the science for adeno-associated virus integration: An integrated perspective. Mol. Ther. *30*, 2646–2663. https://doi.org/10.1016/j.ymthe.2022.06.004.
31. Schmidt, M., Foster, G.R., Coppens, M., Thomsen, H., Cooper, D., Dolmetsch, R., Sawyer, E.K., Heijink, L., and Pipe, S.W. (2021). Liver safety case report from the phase 3 HOPE-B Gene therapy trial in adults with hemophilia B [abstract]. Res. Pract. Thromb. Haemost. *5* (*Suppl 2*). https://abstracts.isth.org/abstract/liver-safety-case-report-from-the-phase-3-hope-b-gene-therapy-trial-in-adults-with-hemophilia-b/.
32. Oziolor, E.M., Kumpf, S.W., Qian, J., Gosink, M., Sheehan, M., Rubitski, D.M., Newman, L., Whiteley, L.O., and Lanz, T.A. (2023). Comparing molecular and computational approaches for detecting viral integration of AAV gene therapy constructs. Mol. Ther. Methods Clin. Dev. *29*, 395–405. https://doi.org/10.1016/j.omtm.2023.04.009.
33. Sato, H., Niimi, A., Yasuhara, T., Permata, T.B.M., Hagiwara, Y., Isono, M., Nuryadi, E., Sekine, R., Oike, T., Kakoti, S., et al. (2017). DNA double-strand break repair pathway regulates PD-L1 expression in cancer cells. Nat. Commun. *8*, 1751. https://doi.org/10.1038/s41467-017-01883-9.
34. Cuartas, J., and Gangwani, L. (2022). R-loop mediated DNA damage and impaired DNA repair in spinal muscular atrophy. Front. Cell. Neurosci. *16*, 826608. https://doi.org/10.3389/fncel.2022.826608.
35. Gabriel, R., Eckenberg, R., Paruzynski, A., Bartholomae, C.C., Nowrouzi, A., Arens, A., Howe, S.J., Recchia, A., Cattoglio, C., Wang, W., et al. (2009). Comprehensive genomic access to vector integration in clinical gene therapy. Nat. Med. *15*, 1431–1436.
36. Paruzynski, A., Arens, A., Gabriel, R., Bartholomae, C.C., Scholz, S., Wang, W., Wolf, S., Glimm, H., Schmidt, M., and von Kalle, C. (2010). Genome-wide high-throughput integrome analyses by nrLAM-PCR and next-generation sequencing. Nat. Protoc. *5*, 1379–1395. https://doi.org/10.1038/nprot.2010.87.
37. McCarty, D.M., Young, S.M., Jr., and Samulski, R.J. (2004). Integration of adeno-associated virus (AAV) and recombinant AAV vectors. Annu. Rev. Genet. *38*, 819–845. https://doi.org/10.1146/annurev.genet.37.110801.143717.
38. Russell, D.W., Miller, A.D., and Alexander, I.E. (1994). Adeno-associated virus vectors preferentially transduce cells in S phase. Proc. Natl. Acad. Sci. USA *91*, 8915–8919. https://doi.org/10.1073/pnas.91.19.8915.
39. Valdmanis, P.N., Lisowski, L., and Kay, M.A. (2012). rAAV-mediated tumorigenesis: still unresolved after an AAV assault. Mol. Ther. *20*, 2014–2017. https://doi.org/10.1038/mt.2012.220.

YMTHE, Volume *31*

Supplemental Information

Epithelioid neoplasm of the spinal cord in a child with spinal muscular atrophy treated with onasemnogene abeparvovec

Laura Retson, Nishant Tiwari, Jennifer Vaughn, Saunder Bernes, P. David Adelson, Keith Mansfield, Silvana Libertini, Brent Kuzmiski, Iulian Alecu, Richard Gabriel, and Ross Mangum

SUPPLEMENTAL MATERIAL

SUPPLEMENTAL FIGURES

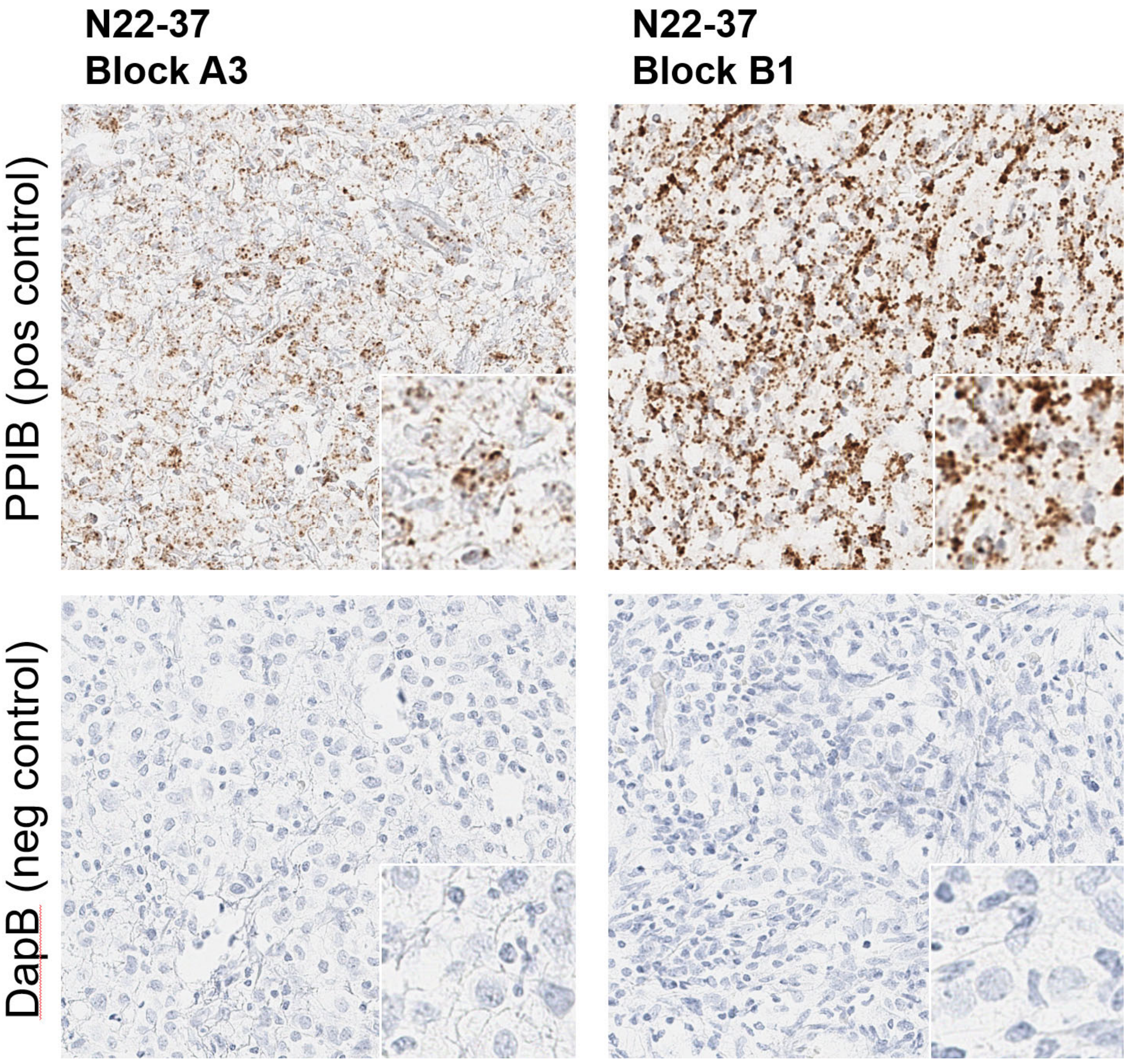

Figure S1. *In situ* hybridization for tissue positive (PPIB) and negative (DapB) controls.
